# Predicting the Impact of Typhoid Conjugate Vaccines on Antimicrobial Resistance

**DOI:** 10.1093/cid/ciy1108

**Published:** 2019-03-07

**Authors:** Samantha Kaufhold, Reza Yaesoubi, Virginia E Pitzer

**Affiliations:** 1 Department of Epidemiology of Microbial Diseases, Yale School of Public Health, Yale University, New Haven, Connecticut; 2 Department of Health Policy and Management, Yale School of Public Health, Yale University, New Haven, Connecticut

**Keywords:** transmission dynamics, *Salmonella* Typhi, vaccination, treatment, multidrug resistance

## Abstract

**Background:**

Empiric prescribing of antimicrobials in typhoid-endemic settings has increased selective pressure on the development of antimicrobial-resistant *Salmonella enterica* serovar Typhi. The introduction of typhoid conjugate vaccines (TCVs) in these settings may relieve this selective pressure, thereby reducing resistant infections and improving health outcomes.

**Methods:**

A deterministic transmission dynamic model was developed to simulate the impact of TCVs on the number and proportion of antimicrobial-resistant typhoid infections and chronic carriers. One-way sensitivity analyses were performed to ascertain particularly impactful model parameters influencing the proportion of antimicrobial-resistant infections and the proportion of cases averted over 10 years.

**Results:**

The model simulations suggested that increasing vaccination coverage would decrease the total number of antimicrobial-resistant typhoid infections but not affect the proportion of cases that were antimicrobial resistant. In the base-case scenario with 80% vaccination coverage, 35% of all typhoid infections were antimicrobial resistant, and 44% of the total cases were averted over 10 years by vaccination. Vaccination also decreased both the total number and proportion of chronic carriers of antimicrobial-resistant infections. The prevalence of chronic carriers, recovery rates from infection, and relative fitness of resistant strains were identified as crucially important parameters.

**Conclusions:**

Model predictions for the proportion of antimicrobial resistant infections and number of cases averted depended strongly on the relative fitness of the resistant strain(s), prevalence of chronic carriers, and rates of recovery without treatment. Further elucidation of these parameter values in real-world typhoid-endemic settings will improve model predictions and assist in targeting future vaccination campaigns and treatment strategies.

There are an estimated 12–18 million cases of typhoid fever per year, predominantly occurring in low- and middle-income countries (LMICs) [1, 2]. *Salmonella enterica* serovar Typhi (*S.* Typhi) causes typhoid fever, which may include symptoms ranging in severity from fever and headache to neurological complications and intestinal perforation [3]. The case fatality rate for hospitalized typhoid patients has been estimated to be between 1 and 5%, though it can be as high as 10–20% for patients who are not treated with appropriate antimicrobials [4–6].

In typhoid-endemic settings, clinicians frequently prescribe empiric antimicrobials to patients with suspected typhoid fever. Because the symptoms of many febrile diseases resemble those of typhoid fever, this prescribing practice results in overtreatment [7, 8], which intensifies selective pressure for the development of antimicrobial-resistant (AMR) *S.* Typhi (and other bacteria), and in turn reduces treatment efficacy, leading to increased morbidity and mortality [4]. AMR strains of *S*. Typhi have been spreading around the globe over the past few decades [9], and an extensively drug-resistant strain is currently causing an outbreak in Pakistan [10].

Vaccination may help to prevent typhoid fever, reduce antimicrobial use, and thereby reduce selection pressure and lessen the threat of AMR infections [11]. A reduction in AMR infections was observed following the introduction of pneumococcal conjugate vaccines (PCVs) [12, 13]. Typhoid conjugate vaccines (TCVs) may have a similar effect. Due to the demonstrated efficacy and sustained immunogenicity in children as young as 6 months, TCV use in typhoid-endemic countries was recently recommended by the World Health Organization’s (WHO) Strategic Advisory Group of Experts [6, 14, 15]. Countries with a high burden of AMR *S.* Typhi are prioritized for vaccine introduction [6].

Implementation of the WHO recommendations for TCV use will require local- and country-level policy and funding decisions that can be informed by models. Previous studies have suggested that TCVs would be cost-effective in some LMIC settings [16, 17]. To further inform these decisions, we examined the impact of TCVs on AMR prevalence and incidence using mathematical models. Both treatment-induced acquired resistance and transmitted AMR infections were considered. Our primary hypothesis was that increased TCV coverage in typhoid-endemic settings would cause a reduction in the prevalence and incidence of AMR typhoid cases. Importantly, we undertook sensitivity analyses to identify critical gaps in data required to inform such a perspective, which in turn can help to prioritize future research.

## METHODS

### Model Description

**Figure 1.**
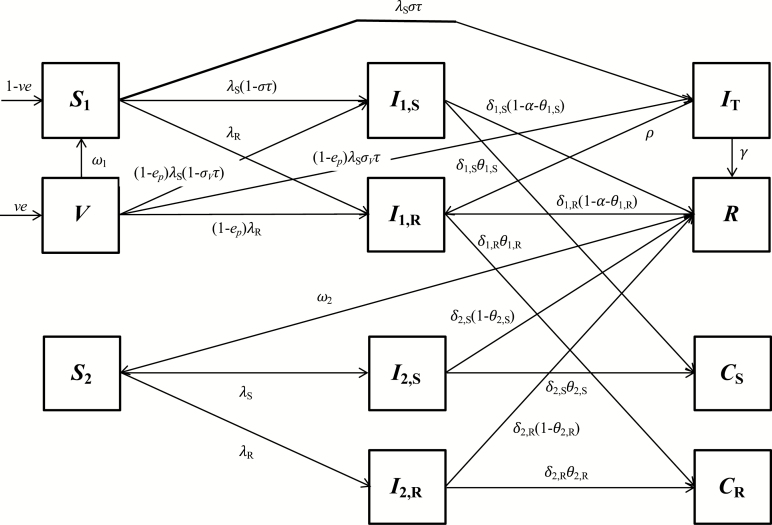
Compartmental structure of transmission dynamic model. The states and parameters are defined in the “Model description” section, while the differential equations for the model are in the Supplementary Material. Natural mortality from each compartment (at rate *μ*) was excluded from the diagram for visual clarity.

A compartmental model of endemic typhoid transmission was modified to include 2 mechanisms of antimicrobial resistance: transmission of resistant *S.* Typhi strains and treatment-induced acquired resistance [18]. The model structure is shown in Figure 1. Individuals are assumed to be born either susceptible to infection (*S*_1_) or into a vaccinated state (*V*). The vaccination process is intended to mimic routine immunization of susceptible infants prior to their first exposure to *S.* Typhi. A fraction *e* of vaccinated individuals is protected, and protection wanes at a rate *ω*_1_. We assume equal protection against antimicrobial-sensitive and AMR strains. Protection from vaccination may be partial, such that the rate of infection is reduced by a factor 1-*e*_*p*_ and the probability of symptoms given infection is *σ*_*V*_ among vaccinated individuals. Susceptible individuals can be infected by *S.* Typhi strains that are initially sensitive (*I*_1,S_, at a rate *λ*_S_) or resistant to first-line antimicrobials (*I*_1,R_, at a rate *λ*_R_). A fraction (*στ*) of susceptible individuals infected by antimicrobial-sensitive *S.* Typhi develop symptoms and are treated, and treated individuals (*I*_T_) either recover (*R*, at a rate *Υ*) or develop treatment-induced acquired resistance (and enter *I*_1,R_ at a rate *ρ*). We assume treatment does not shorten the infectious period of individuals initially infected by AMR strains. Individuals with primary infections of both types remain infectious for a given time (1/*δ*_1,S_ or 1/*δ*_1,R_ depending on the infection type), after which a fraction (*θ*_1,S_ or *θ*_1,R_) develops chronic infections of the gallbladder and become chronic carriers (*C*_*S*_ or *C*_*R*_), a fraction (*α*) experience disease-induced mortality, and the remainder recover, becoming temporarily immune (*R*). After immunity wanes (at a rate *ω*_2_), individuals become susceptible to subclinical reinfection, again by either antimicrobial-sensitive (*I*_2,S_, at a rate *λ*_S_) or AMR *S.* Typhi (*I*_2,R_, at a rate *λ*_R_). Subclinically infected individuals could either become chronic carriers or recover, although the fractions that become chronic carriers following subclinical infections were set to zero in accordance with other typhoid models [16]. Additionally, individuals in all states are subject to natural mortality (at a rate *μ*). We assume the infectiousness of chronic carriers is reduced compared to individuals with primary infections due to intermittent shedding (by a factor *r*_*C*_, which generates a level of indirect protection from vaccination consistent with observations [16, 18]), and the infectiousness of resistant strains is reduced or enhanced compared to antimicrobial-sensitive strains (by a factor *r*_*R*_). The ordinary differential equations describing the model are provided in the Supplementary Materials.

We modeled a hypothetical cohort of 1 000 000 individuals with demographic information contrived to reflect a typical typhoid-endemic setting. The fixed model parameters are provided in Table 1. The remaining model parameters were assigned a base-case value and plausible range (Table 2), which we further examined using 1-way sensitivity analyses.

**Table 1.** Fixed Model Parameters

Parameter Description	Symbol	Value	Source
Population size	N	1 000 000	Assumption
Birth and death rate	*μ*	25 live births (or deaths) per 1000 people per year	Assumption
Duration of infectiousness for first antimicrobial-sensitive infection (without treatment)	1/*δ*_1,S_	4 weeks	[19, 20]
Fraction who experience disease-induced mortality	*α*	.005	[5, 21]
Duration of temporary immunity following recovery from natural infection	1/*ω*_2_	104 weeks	[20, 22]

**Table 2.** Varied Model Parameter Values

Parameter Description	Symbol	Base Case	Range	Source
Transmission parameter	*β*	.3	.05–.5	[16]
Vaccine efficacy (initial)	*e*	.95	.8–1	[15, 16, 23, 24]
Reduction in risk of infection for vaccinated (and protected) individuals	*e*_*p*_	1	.5–1	[25]
Duration of protection from vaccination	1/*ω*_1_	19.2 years	5–25 years	[16, 24]
Fraction symptomatic	*σ*	.2	.1–1	[16]
Fraction symptomatic for vaccinated individuals	*σ*_*V*_	.05	0–.2	[15, 23, 25]}
Fraction treated	*τ*	.75	0–1	[16, 26]
Duration of infectiousness with treatment	1/*Υ*	1 week	.5–3 weeks	[19]
Rate of treatment-induced/acquired resistance	*ρ*	.1 per week	.01–2 per week	Assumption
Natural recovery rate from first, antimicrobial-resistant infection	*δ*_1,R_	.2 per week	.1–.5 per week	[19]
Relative infectiousness of resistant strain(s)	*r*_*R*_	.9	.5–2	Assumption, [27]
Relative infectiousness of chronic carriers	*r*_*C*_	.35	.1–1	[16]
Fraction that become carriers from first, antimicrobial-sensitive infection	*θ*_1,S_	.03	.003–.1	[28]
Fraction that become carriers from first, antimicrobial-resistant infection	*θ*_1,R_	.03	.003–.1	[28]
Relative rate of recovery from second infection	*δ*_2,S_/*δ*_1,S_ = *δ*_2,R_/*δ*_1,R_	1	.5–2	Assumption

### Determination of TCV Impact

We simulated the cohort’s movement through the model compartments for 15 years after a burn-in period of 100 years. Routine vaccination was introduced beginning in year 5, with 5 vaccine coverage scenarios considered: no vaccination, 30% coverage, 50% coverage, 80% coverage, and 100% coverage. The population-level coverage thus increased over time as vaccinated cohorts aged.

The proportion of AMR infections was calculated for each scenario by dividing the number of AMR cases by the total number of cases. The number of cases averted over the 10-year vaccination period was calculated by subtracting the total cases in each vaccination coverage scenario from the number of cases that occurred without vaccination. Additionally, the prevalence of chronic carriers of both antimicrobial-sensitive and AMR strains were calculated by dividing the number of carriers by the total population size.

### Sensitivity Analysis

We performed 1-way sensitivity analyses by varying the parameters one at a time to the minimum and maximum values of the ranges given in Table 2 while holding all other parameters fixed at their base-case values. We quantified the impact of each parameter on the proportion of infections that were AMR at the end of the simulation and the percent of total cases averted by vaccination over 10 years, assuming 80% coverage.

## RESULTS

**Figure 2.**
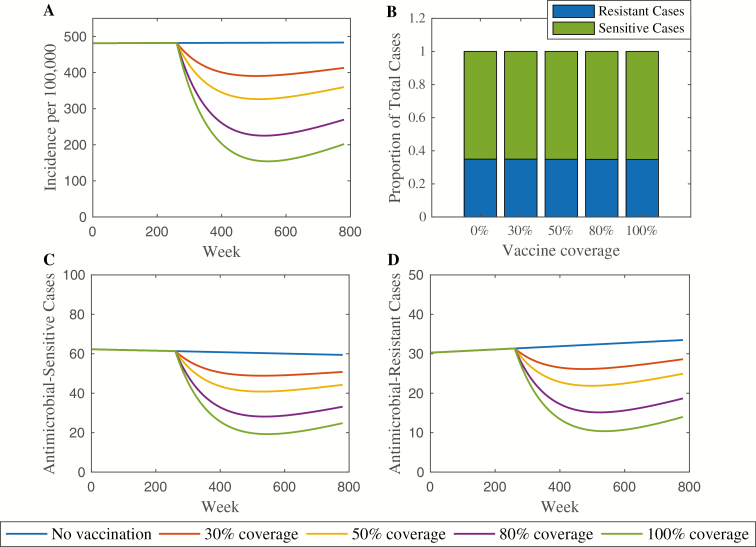
Base-case model simulation. The annual incidence rate per 100 000 person-years is shown in panel *A*. Under each vaccination coverage scenario, the number of antimicrobial-resistant (AMR) infections was divided by the total number of cases after 15 years. In all scenarios, the proportion remained constant at .35, as shown in panel *B*. The total number of antimicrobial-sensitive cases is shown in panel *C*, with AMR cases in panel D. Vaccination was introduced in year 5 (week 260).

The base-case model produced an annual incidence rate between 200 and 500 cases per 100 000 individuals per year depending on vaccination coverage, with the prevalence of chronic carriers varying between 2.2% and 2.6%. For comparison, the incidence rate of typhoid fever across southern Asia has been approximated as 100–1000 cases per 100 000 person years, and recent estimates place the chronic carriage rate at 2–5% in typhoid-endemic settings [29, 30]. The base-case simulation is depicted in Figure 2. The annual incidence rate of typhoid decreases with increasing vaccination coverage (Figure 2A). Without vaccination, the annual incidence rate at the end of the 15-year period is 483 cases per 100 000 person-years. The incidence rate drops to 270 cases per 100 000 person-years with 80% coverage (44% decrease) and 202 cases per 100 000 person-years with 100% coverage (58% decrease). In each vaccination scenario, typhoid incidence declines directly following vaccine introduction. However, the incidence rebounds somewhat 4–5 years later. This reflects the fact that indirect protection afforded by vaccination delays typhoid infections in an endemic setting rather than providing protection from ever being infected.

The number of antimicrobial-sensitive and AMR typhoid cases decreases with increasing vaccination coverage (Figures 2C–D). However, the proportion of AMR cases among the total number of cases remains constant across the 5 vaccination scenarios. Using the base-case model parameter values, the proportion of resistant cases after 15 years is .35 regardless of increasing vaccination coverage (Figure 2B).

**Figure 3.**
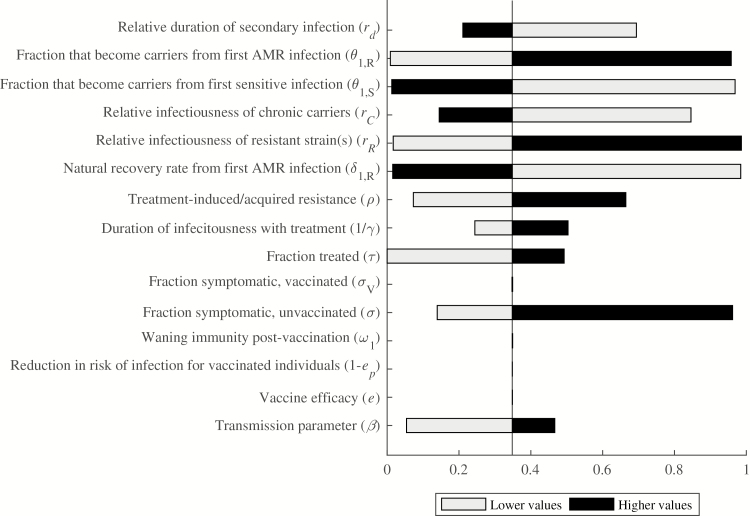
One-way sensitivity analyses for proportion of resistant infections. Parameter values were varied individually to assess their impact on the proportion of AMR cases after 15 years. The comparator proportion of resistant infections was .35, using the base-case parameter values. Black bars represent higher parameter values, whereas grey bars represent lower parameter values. The horizontal axis shows the proportion of AMR cases, with possible values ranging from 0 to 1. Abbreviation: AMR, antimicrobial-resistant.

The proportion of cases that are AMR varied from 0 to .99 depending on the parameter values assessed in one-way sensitivity analyses (Figure 3). The parameters with the most influence on the proportion of AMR cases are the relative infectiousness of resistant strain(s), recovery rate from AMR infection, and the fractions that become chronic carriers from both sensitive and resistant primary infections. The proportion of AMR cases varies from .017 to .986 as the relative infectiousness of the resistant strain(s) increases from .5 to 2, and varies from .984 to .016 as the recovery rate from primary AMR infection increases from .1 to 1 per week. The proportion of AMR cases also increases (from .009 to .958) as a higher fraction of the population becomes chronic carriers from AMR infections. The opposite effect holds for the fraction that become chronic carriers from antimicrobial-sensitive infections; as this fraction varies from .003 to .1, the proportion of AMR cases decreases from .969 to .013.

The other parameters investigated were less impactful on the proportion of AMR cases. The transmission rate, rate of treatment-induced/acquired resistance, fraction symptomatic, fraction treated, and duration of infectiousness with treatment were all directly related to the proportion of AMR cases. Conversely, the proportion of AMR cases decreased as the relative infectiousness of chronic carriers and the relative rate of recovery from secondary infections increased. Notably, none of the vaccine efficacy parameters had an impact on the proportion of AMR infections, nor did the duration of protection conferred by vaccination.

### Vaccine Impact on Percentage of Cases Averted

**Figure 4.**
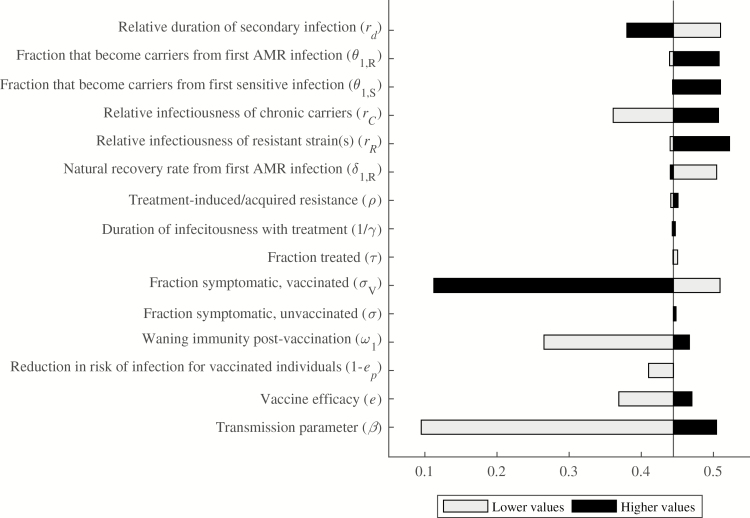
One-way sensitivity analyses for the proportion of cases averted by vaccination. Parameter values were varied individually to assess their impact on the proportion of total cases averted in the 10 years following vaccine introduction. The comparator proportion of cases averted was .44, using the base-case parameter values. Black bars represent higher parameter values, whereas gray bars represent lower parameter values. The horizontal axis shows the proportion of cases averted, with possible values ranging from 0 to 1. Abbreviation: AMR, antimicrobial-resistant.

In the base-case model, 80% vaccination coverage resulted in 21 449 cases averted over 10 years following vaccine introduction, which represents a 44% reduction compared to no vaccination. Because cases averted are of paramount interest to public health practitioners and policymakers, 1-way sensitivity analyses were also used to examine this result. The percent of cases averted by vaccination varied from 9.5% to 52.2%, while the number of cases averted varied from 1069 (when we assumed the transmission rate was .05) to 115 950 (when we assumed all primary infections were symptomatic). The most impactful parameters on the percent of cases averted were the transmission rate and proportion of cases among vaccinated individuals that are symptomatic, followed by the duration of immunity afforded by vaccination, and the relative infectiousness of chronic carriers (Figure 4).

As the transmission parameter (*β*) increased from .05 to .5, the typhoid incidence rate in the absence of vaccination varied from 111 to 505 cases per 100 000 person-years, and the percent of cases averted rose from 9.5% to 50.4%. If we assumed that vaccination only provides partial protection, reducing the risk of infection by 50%, and breakthrough infections among vaccinated individuals were equally likely to be symptomatic (*σ*_*V*_ = .2), then vaccination was only predicted to avert 11.2% of cases; however, if breakthrough infections among vaccinated individuals were asymptomatic (*σ*_*V*_ = 0), vaccination was predicted to avert 50.9% of cases. The proportion of cases that were symptomatic among unvaccinated individuals had a large impact on the number of cases averted but not on the percent of cases averted. The vaccine efficacy, duration of vaccine-induced immunity, relative infectiousness of chronic carriers, and relative rate of recovery from secondary infections also influenced the percent of cases averted by vaccination; the first 3 parameters were directly proportional to the percent of cases averted, whereas the relative rate of recovery from secondary infections was inversely proportional to the cases averted. The remaining parameters were minimally impactful on the percent of cases averted.

### Vaccine Impact on Chronic Carriers

**Figure 5.**
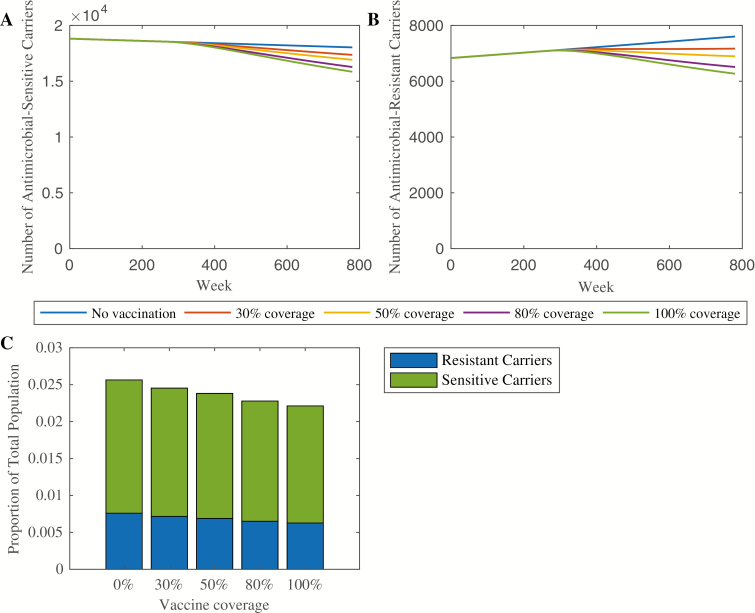
Vaccine impact on chronic carriers. The number of chronic carriers of antimicrobial-sensitive strains throughout the simulation (in a population of 1 million) is shown in panel *A*, whereas the corresponding number of chronic carriers of AMR strains is shown in panel *B*. Panel *C* shows the proportion of the total population which are carriers of both AMR and antimicrobial-sensitive strains under each vaccination scenario at the end of the 15-year simulation. Abbreviation: AMR, antimicrobial-resistant.

Given the importance of chronic carriers to both of the primary endpoints, we also examined the impact of vaccination on the prevalence of chronic carriers. Under the scenario of no vaccination, the number of carriers of antimicrobial-sensitive strains declined throughout the simulation period (Figure 5A). Increasing vaccination coverage caused a sharper decline. By contrast, the number of carriers of AMR strains was increasing throughout the simulation period when vaccine coverage was 30% and below (Figure 5B). Coverage levels of 50% and higher reversed the trend, such that the number of AMR carriers decreased following vaccine introduction. In all vaccination scenarios, the number and proportion of carriers of antimicrobial-sensitive strains was larger than that of AMR strains (Figure 5C), which reflects the more recent emergence of AMR. Increasing vaccination coverage decreased the prevalence of chronic carriers from 2.6% to 2.2%, which may have modest long-term impacts on typhoid transmission.

## DISCUSSION

Our model showed that TCV use has the potential to decrease both total typhoid incidence rates and the number of AMR infections in a hypothetical cohort, but vaccination did not affect the proportion of cases that were AMR. Thus, the number of typhoid cases would need to be decreased to near zero to eliminate AMR strains. The decrease in incidence is consistent with other studies that have shown reductions in both cases and antimicrobial resistance following vaccination for other pathogens. For example, the introduction of the *Haemophilus influenzae* type b conjugate vaccine resulted in substantial declines in both total incidence and the incidence of *β*-lactamase-positive strains [31, 32]. Other studies have also shown a decline in the prevalence of AMR following vaccine introduction. For instance, the introduction of PCV13 resulted in decreased antimicrobial use as well as a decline in the prevalence of AMR pneumococcal strains [13, 33, 34]. Even vaccines for viral diseases like influenza and respiratory syncytial virus, if developed successfully, have the potential to reduce AMR, both by decreasing the likelihood of secondary bacterial infections and by reducing inappropriate prescriptions and resulting antimicrobial use [35]. Therefore, there is reason to believe that TCVs could have a synergistic impact on the prevalence of AMR *S.* Typhi despite our finding that vaccination coverage did not affect the proportion of resistant cases in our model.

One-way sensitivity analyses established that the parameters with the most influence on the proportion of resistant cases were the fraction of individuals that became chronic carriers following infection, the relative infectiousness of resistant strain(s), and the rate of recovery from AMR infection. These findings are consistent with intuition, given the model does not accommodate superinfection with both types of *S.* Typhi strains. A higher fraction of individuals becoming chronic carriers of AMR *S.* Typhi means that more individuals are partitioned off within the model and never recover, thereby perpetually contributing to increased transmission of resistant strains. It is unclear whether AMR strains are more or less likely to lead to chronic carriage of *S*. Typhi. On the one hand, effective antimicrobials have been shown to clear chronic infections with prolonged treatment [36]; thus, failure to clear infection due to AMR should make chronic carriage more likely. However, strains isolated from the gallbladders of chronic carriers have exhibited low rates of antimicrobial resistance [37]. This may simply reflect the more recent emergence of antimicrobial resistance; indeed, our model predicts an increase in the proportion of chronic carriers harboring resistant strains over time in the absence of vaccination.

The relative infectiousness and recovery rate from AMR infection determine the fitness of resistant strains. Strains that are more fit (ie, more infectious and/or with a slower rate of recovery) will result in a higher proportion of resistant cases through an increase in the rate of transmitted resistance. Some fluoroquinolone-resistant *S*. Typhi strains demonstrated a selective advantage over antimicrobial-sensitive strains in the absence of antimicrobial pressure in *in vitro* competition experiments [27]. Furthermore, the H58 haplotype of *S.* Typhi, which is typically associated with antimicrobial resistance, is spreading globally and has replaced antimicrobial-sensitive strains within a matter of years in numerous settings [9, 38, 39], which is suggestive of a fitness advantage.

The proportion of infections that are symptomatic also has a substantial influence on the proportion of cases that are AMR in our model, because we assume symptoms drive treatment-seeking behavior. However, if we assume vaccination only provides partial protection and vary the proportion of infections that are symptomatic among vaccinated individuals, the prevalence of AMR did not change, although the percent of cases averted varied substantially. Nevertheless, if vaccination reduces the prescription and use of antimicrobials to treat typhoid fever beyond the actual reduction in typhoid incidence (eg, due to changes in the perception of typhoid risk), then it is possible this could reduce the prevalence of AMR strains [11].

One-way sensitivity analyses also showed that 2 of the most impactful parameters on the percent of cases averted over 10 years were the typhoid transmission rate and relative infectiousness of chronic carriers. Both parameters lead to a lower average age of infection and hence greater impact of routine immunization. At high transmission rates, the model also predicts a higher prevalence of AMR strains, suggesting that vaccination will be particularly valuable in reducing the number of AMR cases in high-incidence settings. In contrast, antimicrobial-sensitive strains are more prevalent when chronic carriers are more infectious.

Additionally, our analyses suggest that vaccination coverage levels ≥50% can reverse the trend of increasing numbers of chronic carriers of AMR strains. Without effective and prolonged treatment, chronic carriers can remain infectious for the rest of their lives and have been previously identified as important drivers of vaccine impact [18, 40]. Reducing the prevalence of chronic carriers could therefore deplete a key reservoir of antimicrobial resistance, particularly when considering longer time horizons.

As with any simulation study, there were important limitations to our analysis. Crucially, our model did not replicate the conditions of any specific typhoid-endemic setting. It modeled a hypothetical cohort with fixed birth and death rates and was not age-structured. However, the use of a simplified cohort rather than one trained on census data may make these findings more generalizable. Furthermore, the treatment rate was held constant throughout the 100-year burn-in period and 15-year model simulation. Antimicrobial treatment of typhoid fever has only been available since 1948 and has clearly not been used at the same rate for more than 100 years. This assumption may have affected the model outputs, because treatment is a driving factor in the emergence of antimicrobial resistance. Finally, we did not account for introduction of AMR strains from outside the population. Further investigation of the relationship between treatment rates, vaccination, immigration, and the proportion of AMR infections is warranted.

The most important takeaway from our findings is not the proportion of resistant cases produced by base-case parameter values, which are highly uncertain and setting-specific, but the identification of important parameters for further study. The recovery rates, fractions that become chronic carriers, and relative fitness of resistant strains are all natural history parameters that would be particularly useful to characterize in real-world settings. Further elucidation of the relationship between vaccination and treatment could provide crucial information to better target both vaccination campaigns and treatment strategies to limit the spread of AMR typhoid fever.

## Supplementary Data

Supplementary materials are available at *Clinical Infectious Diseases online*. Consisting of data provided by the authors to benefit the reader, the posted materials are not copyedited and are the sole responsibility of the authors, so questions or comments should be addressed to the corresponding author.

Supplementary Material

## Notes

***Financial support.*** This publication is based on research funded in part by a grant from the Bill & Melinda Gates Foundation [OPP1116967, OPP1151153] (V. E .P.), the Wellcome Trust [Strategic Award 106158/Z/14/Z] (V. E. P.), and the National Institutes of Health/National Institute of Allergy and Infectious Diseases [grant 1K01AI119603] (R. Y.).

***Supplement sponsorship.*** This supplement is sponsored by the Center for Vaccine Development and Global Health (CVD) at the University of Maryland School of Medicine.

***Potential conflicts of interest.*** All authors: No reported conflicts. All authors have submitted the ICMJE Form for Disclosure of Potential Conflicts of Interest. Conflicts that the editors consider relevant to the content of the manuscript have been disclosed.
