# Supplementary material for: Predicting the Impact of Typhoid Conjugate Vaccines on Antimicrobial Resistance
Source: Clin Infect Dis. 2019 Mar 7;68(Suppl 2):S96–S104. doi: 10.1093/cid/ciy1108 (PMC6405272; doi:10.1093/cid/ciy1108)
Supplement: Supplementary Material [file ciy1108_suppl_supplementary_material.docx]

**Supplementary Material**

The ordinary differential equations describing the model are as follows:

𝜆_S_ = 𝛽(I_1,S_+I_2,S_+(*r*_C_*C_S_))/N

𝜆_R_ = 𝛽**r*_R_*(I_1,R_+I_2,R_+(*r*_C_*C_R_))/N

$\frac{dS1}{dt}$ = (1-*ev*)𝜇N + 𝜔_1_V - (𝜆_S_ + 𝜆_R_)S_1_ - 𝜇S_1_

$\frac{dV}{dt}$ = *ev*𝜇N - 𝜔_1_V – (1-*e_p_*)(𝜆_S_ + 𝜆_R_) - 𝜇V

$\frac{dI1,S}{dt}$ = 𝜆_S_(1-𝜎𝜏)S_1_ + (1-*e_p_*) 𝜆_S_ (1-*σ_V_τ*)V - 𝛿_1,S_I_1,S_ - 𝜇I_1,S_

$\frac{dIT}{dt}$ = 𝜆_S_𝜎𝜏S_1_ + (1-*e_p_*) 𝜆_S_*σ_V_τ* V - 𝛾I_T_ - 𝜌I_T_ - 𝜇I_T_

$\frac{dR}{dt}$ = 𝛿_1,S_(1-𝛼-𝜃_1,S_)I_1,S_ + 𝛿_1,R_(1-𝛼-𝜃_1,R_)I_1,R_ + 𝛿_2,S_(1-𝜃_2,S_)I_2,S_ + 𝛿_2,R_(1-𝜃_2,R_)I_2,R_ + 𝛾I_T_ - 𝜔_2_R - 𝜇R

$\frac{dI1,R}{dt}$ = 𝜆_R_S_1_ + (1-*e_p_*) 𝜆_R_V - 𝛿_1,R_I_1,R_ + 𝜌I_T_ - 𝜇I_1,R_

$\frac{dCS}{dt}$ = 𝛿_1,S_𝜃_1,S_I_1,S_ + 𝛿_2,S_ 𝜃_2,S_I_2,S_ - 𝜇C_S_

$\frac{dCR}{dt}$ = 𝛿_1,R_𝜃_1,R_I_1,R_ + 𝛿_2,R_𝜃_2,R_I_2,R_ - 𝜇C_R_

$\frac{dS2}{dt}$ = 𝜔_2_R - (𝜆_S_ + 𝜆_R_)S_2_ - 𝜇S_2_

$\frac{dI2,S}{dt}$ = 𝜆_S_S_2_ - 𝛿_2,S_I_2,S_ - 𝜇I_2,S_

$\frac{dI2,R}{dt}$ = 𝜆_R_S_2_ - 𝛿_2,R_I_2,R_ - 𝜇I_2,R_

The model parameters are defined in Tables 1 and 2 of the main text.
